# Knockout Mice Challenge our Concepts of Glucose Homeostasis and the Pathogenesis of Diabetes

**DOI:** 10.1155/EDR.2003.169

**Published:** 2003

**Authors:** C. Ronald Kahn

**Affiliations:** 1 Joslin Diabetes Center Harvard University Medical School Boston Massachusetts USA; 2 Research Division Joslin Diabetes Center One Joslin Place Boston Massachusetts 02215 USA

## Abstract

A central component of type 2 diabetes and the metabolic syndrome is insulin resistance. Insulin exerts a multifaceted and highly integrated series of actions via its intracellular signaling systems. Generation of mice carrying null mutations of the genes encoding proteins in the insulin signaling pathway provides a unique approach to determining the role of individual proteins in the molecular mechanism of insulin action and the pathogenesis of insulin resistance and diabetes. The role of the four major insulin receptor substrates (IRS1-4) in insulin and IGF-1 signaling have been examined by creating mice with targeted gene knockouts. Each produces a unique phenotype, indicating the complementary role of these signaling components. Combined heterozygous defects often produce synergistic or epistatic effects, although the final severity of the phenotype depends on the genetic background of the mice. Conditional knockouts of the insulin receptor have also been created using the Cre-lox system. These tissue specific knockouts have provide unique insights into the control of glucose homeostasis and the pathogenesis of type 2 diabetes, and have led to development of new hypotheses about the nature of the insulin action and development of diabetes.


*Experimental Diab. Res.*, 4:169–182, 2003
Copyright © Taylor and Francis Inc.
ISSN: 1543-8600 print / 1543-8619 online
DOI: 10.1080/15438600390261408

Taylor & Francis
Taylor & Francis Group

**Albert Renold Memorial Lecture**

### Knockout Mice Challenge our Concepts of Glucose Homeostasis and the Pathogenesis of Diabetes

**C. Ronald Kahn**

*Joslin Diabetes Center, Harvard University Medical School, Boston, Massachusetts, USA*

**A central component of type 2 diabetes and the metabolic syndrome is insulin resistance. Insulin exerts a multifaceted and highly integrated series of actions via its intracellular signaling systems. Generation of mice carrying null mutations of the genes encoding proteins in the insulin signaling pathway provides a unique approach to determining the role of individual proteins in the molecular mechanism of insulin action and the pathogenesis of insulin resistance and diabetes. The role of the four major insulin receptor substrates (IRS1-4) in insulin and IGF-1 signaling have been examined by creating mice with targeted gene knockouts. Each produces a unique phenotype, indicating the complementary role of these signaling components. Combined heterozygous defects often produce synergistic or epistatic effects, although the final severity of the phenotype depends on the genetic background of the mice. Conditional knockouts of the insulin receptor have also been created using the Cre-lox system. These tissue specific knockouts have provide unique insights into the control of glucose homeostasis and the pathogenesis of type 2 diabetes, and have led to development of new hypotheses about the nature of the insulin action and development of diabetes.**

**Keywords** Brain; Insulin Action; Insulin Receptor; Insulin Resistance; Knockout Mouse; Mouse Models; Obesity; Type 2 Diabetes; Vascular Endothelium

Over 17 million people in the United States have diabetes mellitus, and about 90% of these have the type 2 form of the disease. In addition, between 17 and 40 million people have insulin resistance, impaired glucose tolerance or the cluster of abnormalities referred to variably as the metabolic syndrome, the dysmetabolic syndrome, syndrome X or the insulin resistance syndrome. In all of these disorders, a central component of the pathophysiology is insulin resistance. In type 2 diabetes there is insulin resistance at the level of the muscle and fat in terms of glucose uptake and insulin resistance at the liver in terms of the ability of the hormone to suppress hepatic glucose uptake (Figure 1). Under normal circumstances, this insulin resistance would be compensated for by increased insulin secreation, but in type 2 diabetes there is a second defect in which the $\beta$-cell specifically loses its ability to sense glucose. This results in insufficient insulin secretion for the level of insulin resistance, i.e. relative insulin deficiency, and a deterioration of glucose tolerance. Insulin resistance is also closely linked to other common health problems, including obesity, polycystic ovarian disease, hyperlipidemia, hypertension and atherosclerosis. Thus, understanding the complex molecular mechanisms of insulin action and its alterations in type 2 diabetes and other insulin resistant states is of critical importance to defining the optimal approach to this group of diseases.

### THE MOLECULAR MECHANISMS OF INSULIN ACTION

Insulin exerts a multifaceted and highly integrated series of actions via its intracellular signaling systems which are schematically represented in Figure 2 [1]. The insulin receptor is a member of the family of receptor, tyrosine kinases. Insulin binds to the $\alpha$-subunit of the receptor, activating the kinase which is intrinsic to the $\beta$-subunit. This results in

Received 22 September 2003; accepted 30 September 2003.
Address correspondence to C. Ronald Kahn, M.D., Research Division, Joslin Diabetes Center, One Joslin Place, Boston, Massachusetts 02215. E-mail: c.ronald.kahn@joslin.harvard.edu

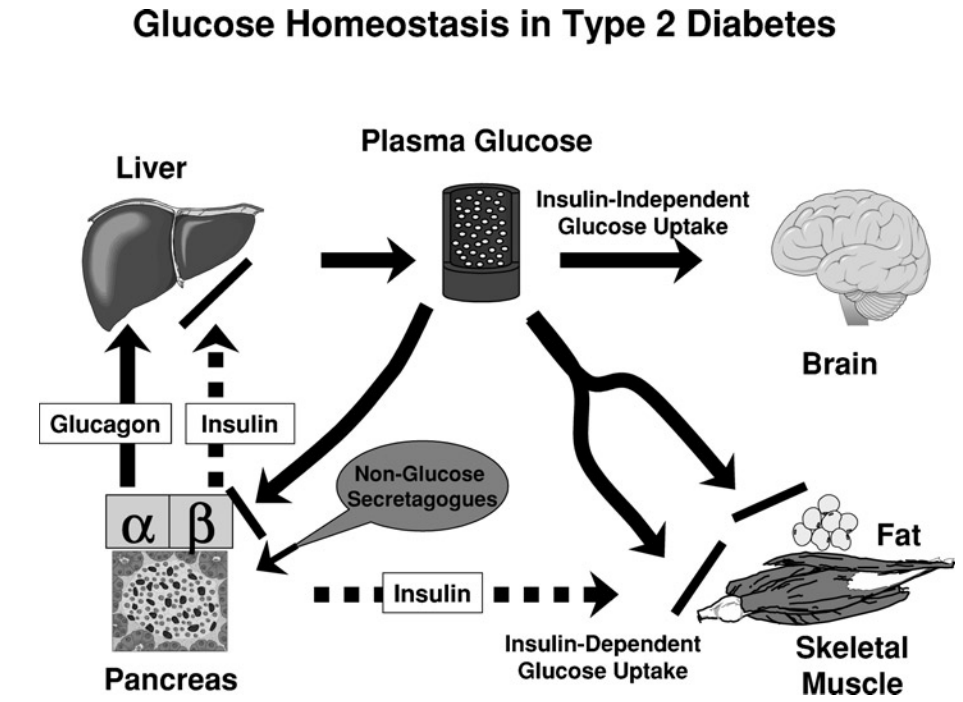

**FIGURE 1**
Control of glucose homeostasis and its alterations in type 2 diabetes.

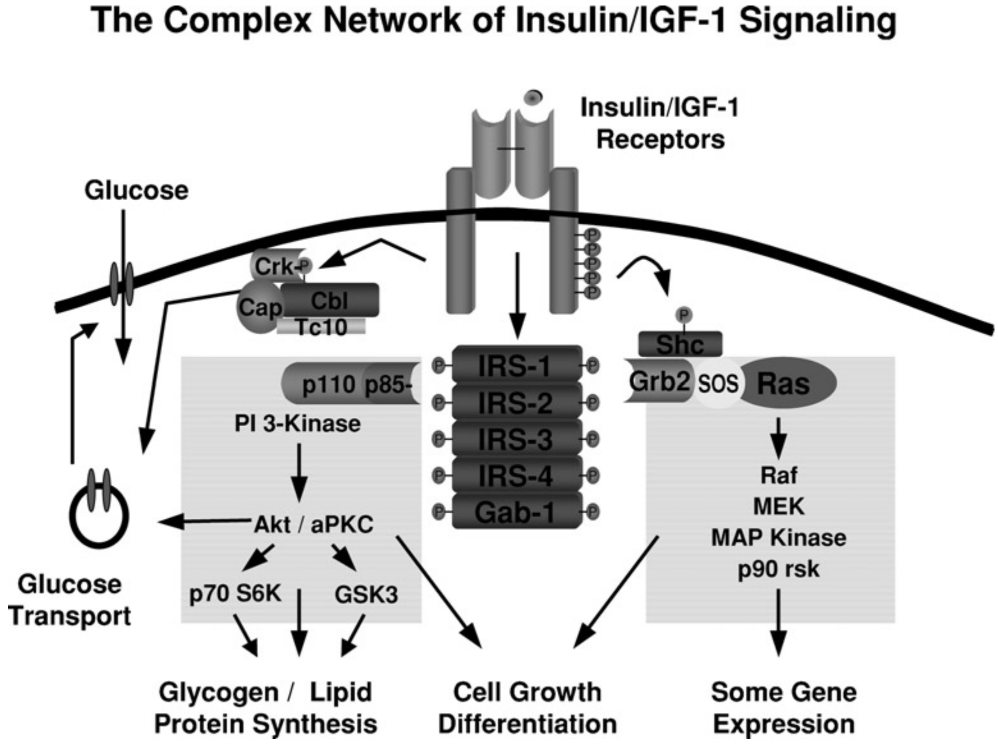

**FIGURE 2**
Schematic overview of the molecular mechanism of insulin action.

autophosphorylation of the receptor itself and futher activation of the receptor toward intracellular substrates. Thus far, ten intracellular substrates of the insulin receptor have been identified. The best characterized of these are the insulin receptor substrate proteins (IRS), IRS-1, -2, -3 and -4 [2–6]. Following tyrosine phosphorylation, each of these intracellular substrates associates with one or more molecules through specific recognition sites terms SH2 domains to generate a downstream signal (Figure 2). The two most important SH2 domain molecules with respect to insulin action are the enzyme phosphatidylinositol (PI) 3-kinase and the adaptor molecule Grb2 [7, 8]. PI 3-kinase appears to be the critical link to all of the metabolic actions of insulin [9], while Grb2 links insulin action to thr Ras-MAP kinase pathway which plays a role in insulin's ability to stimulate cell growth and differentiation [10]. Understanding how this pathway operates and where the defects might be in diabetes and other insulin resistant states is complicated, not only by the multiple IRSs, but also by the fact that each of the proteins involved in insulin action occurs in multiple isoforms. For example, for enzyme PI 3-kinase, there are eight known isoforms of the regulatory subunit and three isoforms of the catalytic subunit. Since at least five of the insulin receptor substrates can link to PI 3-kinase, even in these two steps in the insulin signaling pathway there are 120 potential combinations of signaling proteins that could generate the PI 3-kinase based signal.

### CREATION OF KNOCKOUT MICE

Generation of mice carrying null mutations of the genes encoding proteins in the insulin signaling pathway provides a unique approach to determining the role of individual proteins in the molecular mechanism of insulin action and the pathogenesis of insulin resistance and diabetes. Over the past decade, the function of hundreds, if not thousands, of genes, including many for proteins involved in insulin action, has been analyzed in vivo by gene targeting approaches [11].

The first step to creating global gene knockout is production of a targeting vector containing a modified copy of the gene of interest that is functionally inactive. An example is illustrated in Figure 3. In this case, a clone containing the endogenous gene with the 5′ flanking region, the coding exons and part of the 3′ untranslated region is used to produce a targeting vector, in this example, by deletion of exon 2, which removes the start codon. A neomycin-resistance gene, including a stop codon, is then

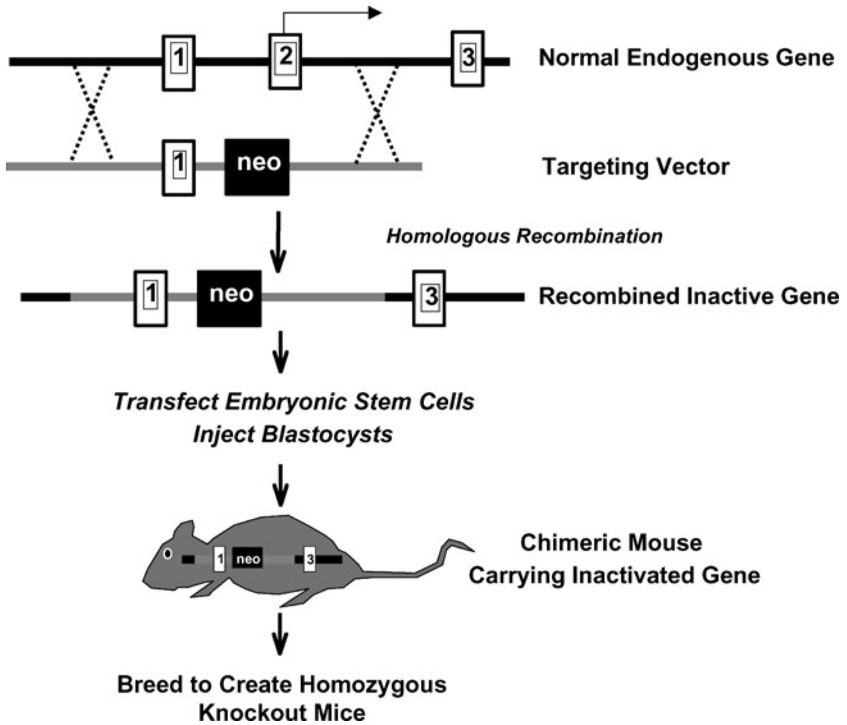

**FIGURE 3**

Gene targeting by homologous recominbination. Homologous recombination between the normal endogenous gene and the targeting vector results in a recombined inactive gene in which exon 2 (containing the start codon) has been substituted by a neomycin cassette (neo). ES cells that survive neomycin selection are injected into blastocysts. Chimeric mice carrying the inactivated gene are being breed to create homozygous knockout mice.

inserted in place of the deleted exon, and the modified gene is transferred, by means of homologous recombination, to replace the normal endogenous genes in embryonic stem (ES) cells. The ES cell clones that survive the neomycin selection can be assessed by Southern blot analysis to confirm that homologous recombination has occurred. This occurs in anywhere between 1 in 2 and 1 in 2,000 cells. The properly targeted cells are then injected into mouse blastocysts, which in turn are implanted into pseudopregnant foster mothers to complete development. The chimeric pups that result can then transmit their modified gene to the progeny. The transmission of the modified gene can be confirmed by PCR and the heterozygous carrier mice can be bred to homogeneity. The absence of the protein can be confirmed by Western blotting of appropriate tissues.

### Insulin Receptor Substrate Knockout Models

Insulin and IGF-1 initiate their actions by stimulating the phosphorylation of a family of proteins referred to as insulin receptor substrates. The role of the four major insulin receptor substrates (IRS-1 to IRS-4) in insulin and IGF-1 signaling have been examined by creating mice with targeted gene knockouts. IRS-1 knockout (KO) mice are IGF-1 resistant and are growth retarded both prenatally and postnatally. They exhibit birth weights between 40–60% of wildtype mice, and this persists throughout adult life [12, 13]. By contrast, IRS-2 knockout mice have only a 10% reduction in birth weight and IRS-3 or IRS-4 knockout mice are of normal size (Table 1) [14–16].

Disruption of *Irs1* also causes insulin resistance, mainly in skeletal muscle, and abnormal glucose tolerance; however, this does not lead to diabetes since these mice can develop $\beta$-cell hyperplasia. The IRS-1 deficiency also leads to other components of the insulin resistance syndrome, such as hypertension and hypertryglyceridemia [17]. Isolated islets from the knockout mice manifest a secretory defect and reduced insulin synthesis, demonstrating a role for IRS-1 in islet function [18]. In contrast, IRS-2 deficient mice develop overt diabetes in early life due to severe insulin resistance in liver and a lack of compensatory hyperplasia from the pancreatic $\beta$-cells [14]. On the other hand, IRS-3 and IRS-4 knockout mice show normal or only minimal defects in glucose homeostasis (Table 1) [15, 16]. This illustrates the unique complementary roles of the IRS-proteins in the insulin/IGF-1 signaling cascades, and the power of mouse genetics to dissect these differences.

**TABLE 1**
Phenotypes of IR and IRS knockout mice

| | |
|---|---|
| Insulin receptor | Normal intrauterine growth<br>Severe insulin resistance<br>Die in 3–7 days in diabetic ketoacidosis |
| IRS-1 | Growth retardation<br>Insulin resistance (muscle, fat)<br>$\beta$-cell hyperplasia |
| IRS-2 | Insulin resistance (Liver)<br>Defect in $\beta$-cell proliferation<br>Diabetes |
| IRS-3 | Normal birth weight<br>Normal glucose homeostasis |
| IRS-4 | Normal growth<br>Very mild defect in glucose homeostasis |
| IRS-1/IRS-2 | Embryonic and fetal lethal |
| IRS-1IRS-3 | Lipoatrophic diabetes<br>Marked insulin resistance |
| IRS-1/IRS-4 | Same as IRS-1 |

### Mice with a Global Knockout of the Insulin Receptor

A complete lack of insulin receptors in humans due to mutations of the insulin receptor gene results in severe insulin resistance and severe intrauterine growth-retardation, but usually only mild to moderate diabetes [19, 20]. Mice with homozygous knockout of the insulin receptor are born at term at the expected mendelian frequency but with only a slight growth retardation ($\sim$10%) and without any apparent metabolic abnormalities [21, 22]. After birth, however, metabolic control rapidly deteriorates: glucose levels increase upon feeding, and insulin levels rise up to 1,000-fold above normal, with no apparent effect on glucose levels. This high level of insulin secretion leads to degranulation of $\beta$-cell cytoplasm and is followed by death of the animals in diabetic ketoacidosis. This phenotype indicates that the insulin receptor is necessary for postnatal fuel homeostasis, but not for embryonic development and metabolic control. A similar phenotype is observed in mice lacking both insulin genes (*Ins1* and *Ins2*) [23].

Interestingly, intraperitoneal administration of IGF-1 causes a prompt and sustained decrease in plasma glucose levels in insulin receptor KO mice, indicating that the glucose lowering effect of IGF-1 occurs through the IGF-1 receptor not the insulin receptor [24]. Despite this "potential compensation," the insulin receptor KO mice die within 7 days after birth.

### TISSUE SPECIFIC KNOCKOUT MOUSE MODELS

Since global gene knockouts may result in a lethal phenotype, conditional knockouts can be created by using tissue specific promoters and thereby allow for a detailed analysis of the gene in a tissue specific manner. For example, the insulin receptor gene has been disrupted in various tissues of the mouse (as described later) by use of the Cre-*loxP* system [25]. This system is based on the use of the bacteriophage recombinase

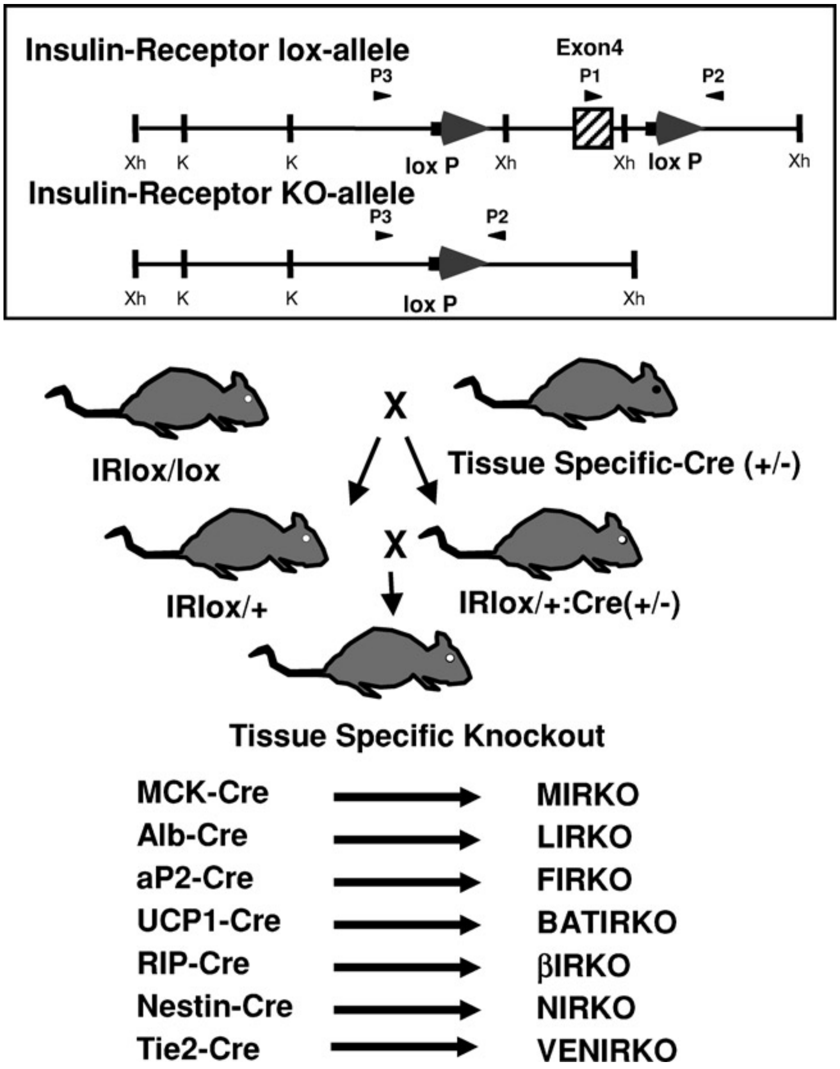

**FIGURE 4**

Schematic representation of the IR/lox allele before and after recombination and mating strategy to generate tissue-specific insulin receptor knockout mice. (Top) The arrows labeled P1, P2, and P3 show the position of the primers used in PCR analyses to confirm recombination. The knockout allele is shown below the floxed allele, indicating the deletion of exon 4 in the event of recombination of the insulin receptor gene [29]. (Lower panel) Strategy for generating tissue-specific insulin receptor knockout mice.

Cre to conditionally inactivate genes in mice in which *loxP* sites have been introduced flanking some critical element. LoxP sites are 34-bp consensus sequences of bacterial DNA, which allow for directional recombination of two segments of DNA, eliminating the DNA which occurs in between (Figure 4). In the case of the insulin receptor, the *loxP* sites were introduced around exon 4, since deletion of this exon in the presence of the Cre recombinase will cause a major deletion and frameshift mutation with a premature stop of translation at amino acid 308 of the insulin receptor protein. Mice carrying the IR*lox* allele can then be bred with mice carrying the Cre recombinase on different promoters to produce a variety of tissue-specific knockouts of the insulin receptor. For example, breeding with an MCK promoter-Cre mouse will produce a muscle-specific knockout of the insulin receptor (MIRKO mouse), albumin-Cre a liver-specific knockout (LIRKO), aP2-Cre (FIRKO), etc. (Figure 4).

### Muscle-Specific Insulin Receptor Knockout (MIRKO) Mice

Since muscle accounts for >80% of post-prandial glucose uptake in humans and is a site of insulin resistance early in the pre-diabetic state [26–28], the first tissue specific knockout to be created was a muscle-specific insulin receptor knockout (MIRKO). As expected, by Western blotting, MIRKO mice demonstrate almost complete and specific ablation of insulin receptor expression in all skeletal muscle, and even a 92% reduction in the heart (which had lower Cre expression) [29]. By contrast, insulin receptor expression was unaltered in other tissues. Mice with a MIRKO were born with the expected frequency and were indistinguishable from their wildtype littermates. Muscle mass was only reduced by ∼10%, and MIRKO mice showed no difference in spontaneous activity or ability to run on a treadmill as compared to controls.

The expectation was that an impairment of insulin signaling in muscle would at least cause systemic glucose intolerance. Surprisingly, despite the virtual lack of insulin signaling in muscle, the MIRKO mice were able to maintain euglycemia up to at least 20 months of age [29]. Moreover, plasma insulin concentrations were indistinguishable from normal. Similarly, these animals were able to clear an intraperitoneal bolus of glucose with same efficiency as control mice (Figure 5*A*) and responded normally to exogenously administered insulin. On euglycemic clamp studies, the rates of insulin-stimulated whole body glucose uptake were decreased by 45% in MIRKO mice with a 74% decrease in insulin-stimulated muscle glucose transport, and there was an 87% decrease in insulin-stimulated muscle glycogen synthesis. Although glucose uptake into muscle was severely decreased in response to insulin, glucose uptake in response to exercise was normal [30].

What was also unexpected, however, was that insulin-stimulated glucose transport in adipose tissue was increased by 3-fold in MIRKO mice (Figure 5*B*). Thus, the relatively normal plasma glucose in MIRKO mice may be due to the ability of exercise to stimulate glucose uptake, as well as a shift of insulin-stimulated glucose uptake and metabolism to adipose tissue. As a result of this substrate shift, MIRKO mice developed a metabolic syndrome with an increased adipose tissue mass and marked hypertriglyceridemia and a modest increase in free fatty acids (Figure 6). These findings (mild obesity, hypertriglyceridemia and elevated FFA) are hallmarks of the metabolic syndrome, Syndrome X.

The MIRKO mice demonstrate the utility and value of tissue-specific knockouts in expanding our insights into the nature of insulin signaling and testing our concepts of glucose homeostasis and the pathogenesis of type 2 diabetes. This mouse also suggests the presence of some cross-talk between muscle and fat, which if it occurs in humans as well, could contribute to the increase in obesity in people with genetically programmed insulin resistance in muscle (Figure 6). Of course, it is possible that the importance of skeletal muscle as a site for glucose disposal has been overestimated, or that in addition to a direct insulin stimulation of glucose uptake into skeletal muscle there could be indirect effects based on increased blood flow across skeletal muscle [31] or generation of locally diffusible mediators of insulin action from the vasculature, such as nitric oxide or cGMP [32, 33] that act on muscle to enhance glucose uptake. Finally, the MIRKO mouse also suggests a significant role of muscle insulin resistance in development of the lipid phenotype of the metabolic syndrome, including elevated plasma triglycerides, FFAs, and central obesity.

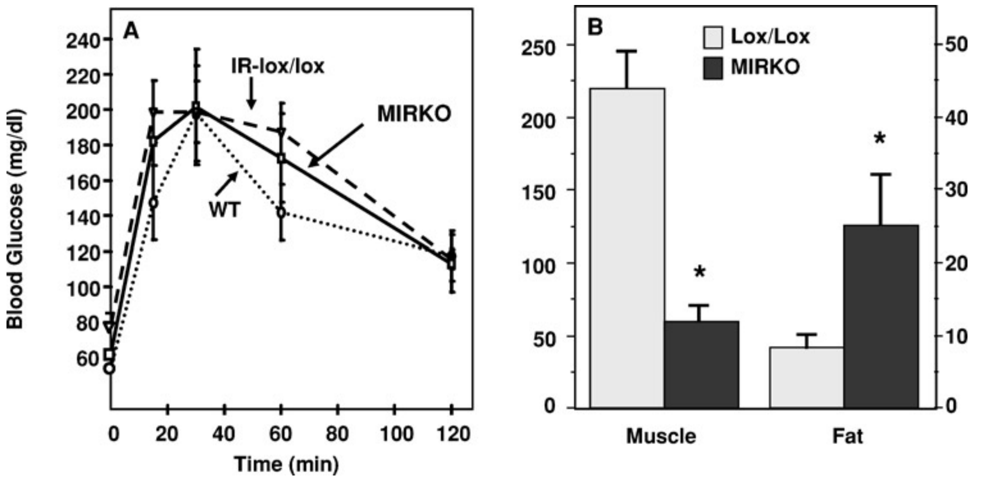

**FIGURE 5**

Physiological consequences of muscle-specific IR gene inactivation. (*A*) Glucose tolerance tests performed on 4-months old wildtype, IR*lox*/*lox* and MIRKO mice. Results are expressed as mean blood glucose concentration ± SEM. (*B*) Glucose uptake into muscle and fat in 4-months old male, IR*lox*/*lox* and MIRKO mice [29]. An star (⋆) indicates $p < 0.05$ for the difference between *lox*/*lox* control and MIRKO.

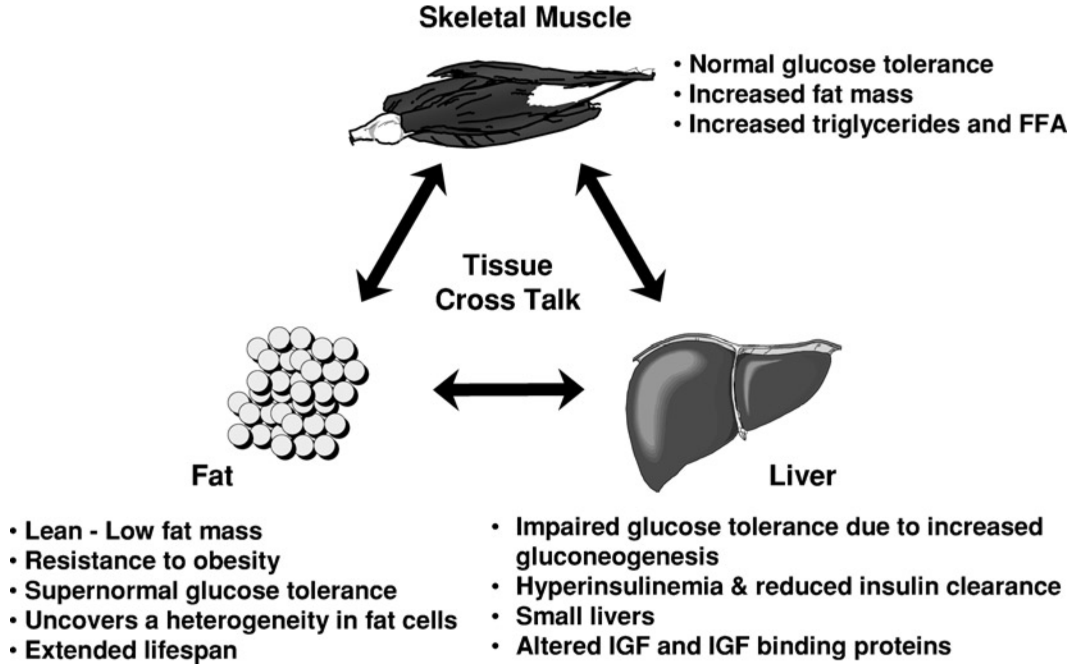

**FIGURE 6**
Defects in muscle, fat and liver-specific insulin receptor knockout mice.

### Fat-Specific Insulin Receptor Knockout (FIRKO) Mice

Although fat tissue accounts for only ~10% of glucose uptake [34, 35], insulin has major effects on adipocytes to promote adipogenesis, stimulate glucose uptake and lipid synthesis and inhibit lipolysis [36]. Mice with a specific knockout of the insulin receptor in both white and brown fat (FIRKO) have been created using the Cre transgene driven by the adipose-specific aP2 promoter [37]. The FIRKO mice were born with the expected frequency, survived well after weaning, and were fertile. These mice have a ~50% decrease in fat pad mass and a ~30% decrease in whole body triglyceride content, with normal circulating lipids, free fatty acids and glycerol. Furthermore, the FIRKO mice are resistant to gaining weight during aging or following induction of a hypothalamic lesion leading to hyperphagia. As a result, these mice not only have normal glucose tolerance, but fail to develop impaired glucose tolerance despite overeating. Thus, it appears that insulin signaling in adipose tissue is not critical for the maintenance of euglycemia in mice, but is required for the development and maintenance of normal triglyceride stores in adipocytes. Other features of the FIRKO mice are inappropriately high leptin levels for fat mass and a polarization of adipocyte cell size with fat pads consisting of large or small fat cells, but very few intermediate sized cells [37].

Interestingly, the FIRKO mice have an increase in mean lifespan of ~134 days, as well as a parallel increase in median and maximum life span (Blueher, Kahn, and Kahn, 2003) (Figure 6). This extended longevity occurred in two independent lines of mice, and in both males and females. Thus, the FIRKO mouse model support studies in *Caenorhabditis elegans* and *Drosophila* showing that insulin-like signaling pathways are involved in regulation of longevity and that leanness and not food restriction is the most beneficial factor on the extension of lifespan (Figure 6) [38–40].

### Brown Adipose-Specific Insulin Receptor Knockout (BATIRKO) Mice

Brown adipose tissue is thought to play an important role in determining peripheral insulin sensitivity [41], as well as thermal adaptation. Insulin receptor has been inactivated in this tissue (BATIRKO) using the uncoupling protein-1 (UCP1) promoter to drive Cre expression. Although brown adipose tissue develops normally in these mice, it undergoes atrophy as the mice age. Surprisingly, the age-dependent loss of brown adipose tissue is associated with an unexpected deterioration of $\beta$-cell function and decrease of $\beta$-cell mass, giving rise to hyperglycemia [42]. This observation suggests that the maintenance of an adequate $\beta$-cell mass some how requires brown adipose tissue. It remains to be determined whether this is an endocrine effect of factors produced in brown adipose tissue, or whether it reflects a broader metabolic change.

### Liver-Specific Insulin Receptor Knockout (LIRKO) Mice

The liver plays a central role in the control of glucose homeostasis and is subject to complex regulation by substrates, insulin and other hormones. The effect of insulin to suppress hepatic glucose output is the result of inhibition of glycolysis initially, but with prolonged fasting depends on the ability of insulin to inhibit gluconeogenesis [43]. Insulin resistance in the liver, and especially the loss of the ability of insulin to suppress hepatic glucose output, are closely correlated with fasting hyperglycemia in type 2 diabetes. In addition, the liver plays an important role in insulin degradation since the clearance of insulin in vivo occurs primarily in the liver and is mediated mainly by receptor dependent mechanisms.

To define the role of the liver in glucose homeostasis, mice were generated with a liver-specific knockout of the insulin receptor (LIRKO) by breeding the mice carrying the *floxed* insulin receptor gene with mice that express Cre recombinase under the albumin promoter/enhancer [44, 45]. The insulin receptor expression and insulin stimulated tyrosine phosphorylation was unaffected in liver from control mice (WT, IR(lox) and Alb-Cre), whereas the insulin receptor expression was reduced by ~95% and insulin receptor autophosphorylation was absent in liver from LIRKO mice (Figure 7*A*). Likewise insulin stimulated tyrosine phosphorylation of IRS-1 and binding of IRS-1 to p85 (the regulatory subunit of PI 3-kinase) in livers from the control mice were similar, while IRS-1 phosphorylation and p85 binding was practically undetectable in LIRKO mice. Although the LIRKO mice are of normal size, for 3–5 days post-weaning, LIRKO mice gain less weight than controls, but this is corrected by 6 weeks of age. On gross examination, at all ages, the livers of LIRKO mice were 50–70% of normal size, and exhibit some morphological changes with collections of large, oval cells.

At 2 months of age, male LIRKO mice were hyperglycemic in the fed sate as compared to controls and exhibited severely

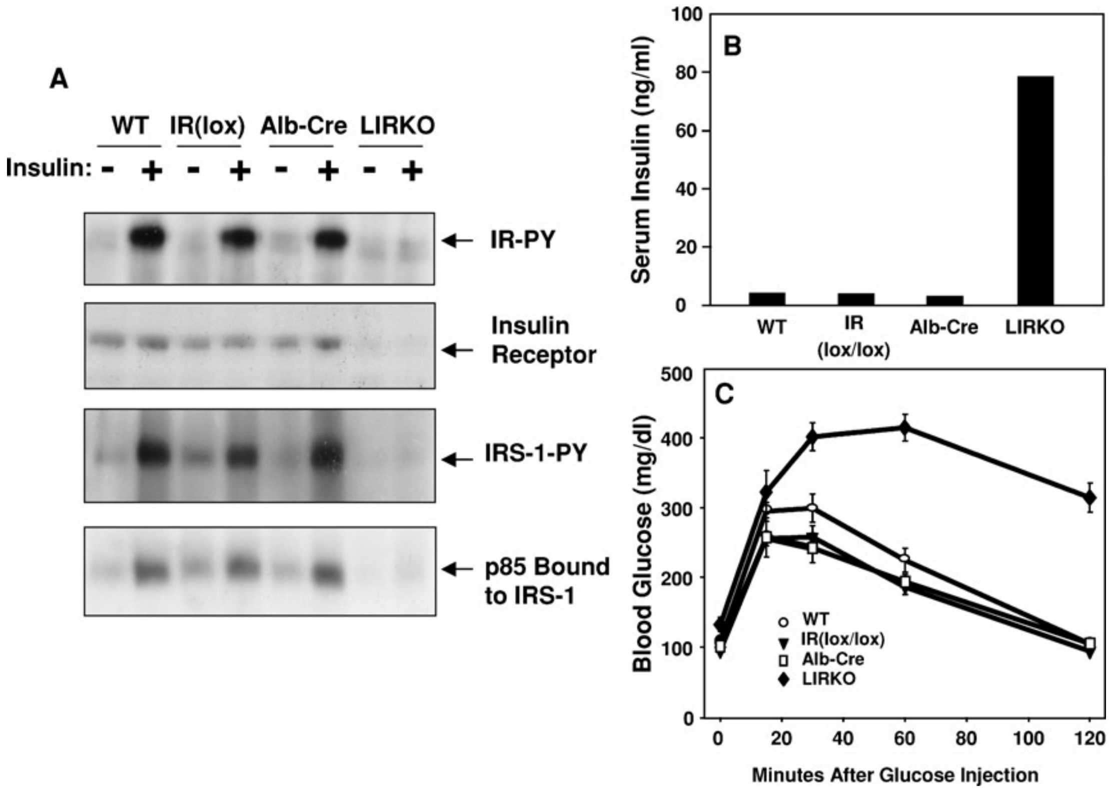

**FIGURE 7**

LIRKO mice have no insulin signaling in liver and a metabolic phenotype of hepatic insulin resistance. (*A*) Basal and insulin stimulated insulin receptor tyrosine phosphorylation, insulin receptor content, IRS-1 tyrosine phosphorylation and p85 binding to IRS-1 in whole livers from control mice and LIRKO mice. (*B*) Fed serum insulin levels. (*C*) Glucose tolerance tests were performed on 2-month-old male wildtype, IR*lox*/*lox*, abl-Cre and LIRKO mice that had been fasted for 16 hours. Animals were injected with 2 g/kg body weight of glucose. Glucose was measured immediately before injection and 15, 30, 60 and 120 minutes after injection. Results are expressed as mean blood glucose concentration ± SEM [44].

impaired glucose tolerance. Serum insulin levels in LIRKO males were elevated by 20-fold in both the fed (Figure 7*B*) and fasting state. Histological analysis also indicated that LIRKO mice have a significant increase in islet size as they attempt to compensate for the insulin resistance. Insulin tolerance tests at 2 months of age demonstrated that LIRKO mice were completely resistant to the blood glucose lowering effects of exogenous insulin. Similarly, glucose tolerance tests performed at 2 months of age demonstrated severe glucose intolerance in LIRKO mice (Figure 7*C*). Somewhat surprisingly, by 4 months of age, the fasting hyperglycemia had disappeared, although fasting and fed hyperinsulinemia, fed hyperglycemia, insulin resistance and glucose intolerance remained. In addition, serum triglycerides and free fatty acids were reduced by 30–50% in 4-month-old male LIRKO mice as compared to control groups [44].

To gain insight into the role of hepatic insulin signaling in the action of insulin sensitizing agents, LIRKO mice were treated with the biguinide metformin and the thiazolidinedione (TZD) rosiglitazone. Although neither rosiglitazone nor metformin altered glucose tolerance or insulin tolerance, LIRKO mice treated with rosiglitazone did exhibit a reduction in their low-density lipoprotein (LDL) cholesterol levels.

Taken together, these studies indicate that isolated liver insulin resistance is sufficient to cause severe defects in glucose and lipid homeostasis, but not uncontrolled fasting hyperglycemia or diabetes (Figure 6). This defect also leads to hyperinsulinemia, due to changes in both insulin secretion and insulin clearance. Furthermore, TZDs may improve some lipid parameters in the LIRKO mice, whereas metformin requires an operating insulin signaling system in the liver.

### Vascular Endothelial Cell Insulin Receptor Knockout (VENIRKO) Mice

Insulin receptors on vascular endothelial cells have been suggested to participate in insulin-regulated glucose homeostasis by facilitating transcytosis of insulin from the intravascular to extracellular space, by promoting vasodilation and enhancing blood flow, and by generation of signaling mediators [46, 47]. To directly address the role of insulin action in endothelial function, mice with a vascular endothelial cell insulin receptor knockout (VENIRKO) were generated using the Cre-*loxP* system (Vicent et al., manuscript submitted). Isolated endothelial cells of VENIRKO mice exhibited complete rearrangement of the insulin receptor gene and a >95% decrease in insulin receptor mRNA. Despite previous studies suggesting a role of the insulin receptor on endothelial cells in control of both glucose homeostasis and vascular tone, blood glucose and insulin concentrations, glucose and insulin tolerance tests, the time course of insulin action on glucose disposal during a euglycemic-hyperinsulinemic clamp were comparable in VENIRKO and control mice. However, both endothelial nitric oxide synthase (eNOS) and endothelin-1 (ET-1) mRNA levels were reduced by 30–60% in endothelial cells, aorta, and heart as assessed by real-time quantitative RT-PCR and Northern blotting, while expression of vascular endothelial growth factor (VEGF) was maintained at normal levels. VENIRKO also tend to have lower systolic, diastolic and mean blood pressures than controls, but respond normally to high and low salt diet. On the low salt diet, however, the VENIRKO mice showed insulin resistance. Thus, inactivation of the insulin receptor on endothelial cell has no major consequences on vascular development or glucose homeostasis under basal conditions, but alters expression of vasoactive mediators and may play a role in maintaining vascular tone and regulation of insulin sensitivity to dietary salt intake.

### Pancreatic $\beta$-Cell-Specific Insulin Receptor Knockout ($\beta$IRKO) Mice

Whether as a result of autoimmune destruction, as in type 1 diabetes, or as a result of impaired function, as in type 2 diabetes, fasting hyperglycemia is inevitably associated with some degree of $\beta$-cell failure. We do not know what the central defect in $\beta$-cells of type 2 diabetics is; however, one of the most characteristic features is an impairment of glucose-stimulated insulin secretion, which is, at least in part, genetically determined [48].

Under normal circumstances, insulin secretion is regulated by the products of glucose metabolism in the $\beta$-cell [49]. However, several observations have raised the possibility that signaling through receptor tyrosine kinases also participates in control of insulin synthesis and release. As described above, a complete knockout of *Irs1* leads to defective insulin secretion in response to glucose and amino acids [18], while inactivation of *Irs2* leads to impaired $\beta$-cell proliferation [14], suggesting a role of insulin action in control of $\beta$-cell function [50].

To directly address the role of insulin signaling in mature $\beta$-cells, mice with the IR*lox* gene were bred with mice carrying the Cre transgene controlled by the rat insulin 2 promoter (Rip-Cre transgenic mice) [51]. The Rip-Cre transgenic mice exhibited specific Cre expression in insulin-producing $\beta$-cells, but not in non-$\beta$-cells or acinar cells [51]. As compared to control mice, $\beta$IRKO mice exhibited an 85% reduction in acute first-phase insulin secretion in response to glucose and virtually no response in males. In contrast, the acute insulin release in response to arginine was maintained. Immunohistochemistry of the islets showed no differences in islet size or in the ratio of $\beta$ to non-$\beta$-cells at 2 months of age, and insulin content was normal. In 4-month-old control mice, islet size and insulin content had increased slightly, but this did not occur in the $\beta$IRKO mice.

Upon intraperitoneal glucose challenge, $\beta$IRKO mice showed significantly higher glucose levels than in the controls, and the glucose tolerance continued to worsen with time [51]. Thus, a lack of insulin receptors in $\beta$-cells results in a selective impairment of glucose-dependent insulin release, and leads to an age-dependent glucose intolerance and, in some mice, overt diabetes [51]. From these data, it appears that signaling through receptor tyrosine kinases regulates both $\beta$-cell proliferation and insulin secretion.

It is unclear how insulin controls its own secretion and more work is required to determine the kinetics of insulin regulation of its own secretion. Nevertheless, the idea that insulin receptor signaling regulates insulin production and exocytosis is teleologically attractive, in that it would provide a unifying mechanism for insulin resistance and impaired $\beta$-cell function. Although, the insulin-receptor related receptor (IRR), is also expressed in $\beta$-cells [52], IRR does not appear to be involved in this process. In fact, metabolic analyses and insulin release studies from perifused islets of IRR knockouts have failed thus far to demonstrate a role for this receptor in $\beta$-cell function [53].

### Neuron-Specific Insulin Receptor Knockout (NIRKO) Mice

Insulin and IGF1 receptors are expressed at high levels in many brain areas and different cell types, including glial and neuronal cells [54]. Since neurons metabolize glucose in an insulin-independent manner, the role of insulin receptor in the brain has remained somewhat unclear. To directly assess the role of insulin in brain, a neuron-specific insulin receptor knockout (NIRKO) was generated using nestin/Cre-mediated ablation [55]. NIRKO mice showed a >95% reduction in the level of insulin receptor in the brain. The NIRKO mice showed increased food intake and moderate diet-dependent obesity associated with insulin resistance and hypertriglyceridemia. NIRKO mice also exhibited hypogonadotropic hypogonadism, associated with impaired maturation of ovarian follicles in females and reduced spermatogenesis in males, leading to reduced fertility [55]. Thus, insulin receptors in brain play a role in the control of appetite reproduction. Recent studies suggest that this occurs through an effect of insulin on neuropeptide Y (NPY) and orexin expression (Bruning, personal communication). Other work has indicated that inhibition of insulin receptors by antisense oligonucleotides and blocking antibodies affects signaling through the melanocortin pathway [56].

### DIFFERENT SITES OF INSULIN RESISTANCE PRODUCE DIFFERENT PHENOTYPES

### Muscle Specific GLUT4 Knockout (MG4KO) Mice

GLUT4 is referred to as the insulin-sensitive glucose transporter since it is mainly expressed in skeletal muscle, heart and adipose tissue [27], and mediates glucose transport stimulated by insulin and contraction/exercise [57]. To determine if different types of insulin resistance in a single tissue might produce different alterations in glucose homeostasis, the Cre/*loxP* sytem was used to selectively disrupt GLUT4 expression in muscle [58]. To this end, a construct with *loxP* sites flanking exon 10 (encoding the glucose binding site) of the mouse GLUT4 gene was introduced into mice using homologous recombination, and the resultant mice were bred to the MCK-Cre mice as described for the MIRKO mouse. The muscle-specific GLUT4 knockout (MG4KO) mice had >90–95% reduction of GLUT4 protein in skeletal muscle and no compensatory increase in GLUT1. Growth curves of the MG4KO mouse were normal until 6 months of age, after which they tended to gain weight more slowly [58]. Basal glucose uptake in vitro was reduced 72% in soleus and 88% in EDL muscles of MG4KO mice compared to controls and the response to insulin was completely obliterated. Fasting glucose was higher in MG4KO mice by 8 weeks compared to control, and intraperitoneal glucose tolerance tests were significantly impaired. Intraperitoneal insulin injection and contraction produced no fall in glucose levels in male MG4KO and only a 34% fall in female MG4KO ($p < .03$). Hyperinsulinemic-euglycemic clamp studies of the mice showed a 55% decrease in insulin-stimulated whole body glucose uptake in MG4KO mice as compared to wildtype mice demonstrating a severe insulin resistance in the knockout [59]. The decrease in whole-body glucose uptake could mainly be attributed to a reduction by 92% in the insulin-stimulated glucose uptake in skeletal muscle of the MG4KO [59]. Thus, in contrast to the MIRKO mice, the MG4KO mice have severe whole body insulin resistance, fasting hyperglycemia and glucose intolerance, but show no increase in body fat or associated hyperlipidemia.

### Fat Specific GLUT4 Knockout Mice

Fat-specific GLUT4 knockout mice were created by crossing Glut4 *loxP* mice with mice expressing the Cre recombinase under the control of the aP2 promoter/enhancer [60]. GLUT4 expression in the resulting mice was markedly reduced in both brown and white adipose tissue, whereas GLUT1 expression was normal and the mice showed growth curves similar to control mice. The basal glucose uptake into adipocytes tended to be reduced by about 40%, and the maximal insulin stimulated glucose uptake was reduced by 72% in the knockout mice compared to control mice. In contrast, the basal and insulin stimulated glucose transport into skeletal muscle was unaltered. In vivo studies performed during a hyperinsulinemic-euglycemic clamp of the GLUT4 fat-specific knockout mice showed a 53% decrease in insulin-stimulated whole body glucose uptake and reductions in glycolysis and glycogen synthesis by 50–67% [60]. Insulin-stimulated glucose transport into white and brown adipose tissue was markedly reduced and surprisingly, the glucose transport into skeletal muscle was impaired by 40%, despite the preserved expression of GLUT4 in muscle. This discrepancy between in vivo and in vitro muscle glucose uptake is suggested to be due to secondary defects in the in vivo milieu resulting from altered release of specific molecules from fat [60]. Furthermore, a secondary effect of the GLUT4 fat-specific knockout was also detected in the liver, in which the ability of insulin to suppress hepatic glucose production was decreased as compared to control mice. Thus, the insulin resistance created in fat due to GLUT4 deficiency causes secondarily induced insulin resistance in other insulin target tissues. The insulin resistance created by insulin receptor knockout in fat (FIRKO) also causes a decrease in insulin stimulated glucose uptake into white adipose tissue [37], however in contrast to the GLUT4 fat-specific knockout mice, the FIRKO mice are protected from age-related reduction in whole body glucose and insulin tolerances. Thus, insulin resistance in fat at the level of the insulin receptor versus GLUT4 produce dramatically different metabolic outcomes on both fat and at the whole-body level.

### POLYGENIC KNOCKOUT MODELS

### Mice with Compound Defects Mimic Human Type 2 Diabetes

Since type 2 diabetes is polygenic in nature, a heterozygous doubleknockout mouse model of insulin receptor and IRS-1 has been generated to create a polygenic model of diabetes that may more closely mimic the human disease. This was possible since heterozygote insulin receptor and heterozygote IRS-1 knockout mice exhibit only mild subclinical insulin resistance with a 1.5- to 2-fold elevation in insulin levels and mild $\beta$-cell hyperplasia. In contrast, the IR/IRS-1 doubleheterozygous (DH) knockout mice manifested marked insulin resistance with 10-fold increases in circulating insulin levels and a 5–30 fold increase in $\beta$-cell mass. Despite this islet hyperplasia on a mixed genetic background, ~50% of these mice developed diabetes by 4–6 months of age [61].

These compound heterozygote animals revealed several features of interest. First, despite the genetic nature of the insulin resistance, like humans, these mice develop diabetes with delayed onset. Secondly, the development of diabetes indicates a marked synergism (epistasis) between the insulin receptor defect (which leads to diabetes in <10% of mice) and the IRS-1 defect (which never leads to diabetes). Finally, on a mixed genetic background only ~50% of the mice develop diabetes, even after up to 18 months of follow-up, indicating that an additional gene or genes present in the background of these mice contribute to or protect from the development of diabetes.

Recent experiments have shown that the phenotype of male mice DH for the insulin receptor and IRS-1 varies markedly depending on the genetic background. Thus, DH mice on the C57Bl/6J (B6) background have a high incidence of diabetes (85% at 6 months of age), whereas the 129Sv strain does not develop diabetes, despite the genetic defects in insulin signaling (Figure 8). This difference is due to differences in insulin resistance, rather that $\beta$-cell failure.

In order to identify the susceptibility or resistance alleles we created an F2 intercross between mice on the B6 and 129Sv backgrounds. The incidence of diabetes in DH male intercross mice was 60% at 6 months of age and fed blood glucoses showed a wide variation and a bimodal distribution. Likewise, the fed plasma insulin levels also exhibited a wide range. The relationship between fed insulin and glucose levels of the DH mice described a bell-shaped curve, as has been observed in several studies of humans with type 2 diabetes [62] and some rodent models [14]. At any glucose, however, there was a wide range of insulin values suggesting a wide range of insulin resistance in the DH mice. Thus, the IR/IRS-1 DH knockout mouse is similar to human type 2 diabetes with a polygenic

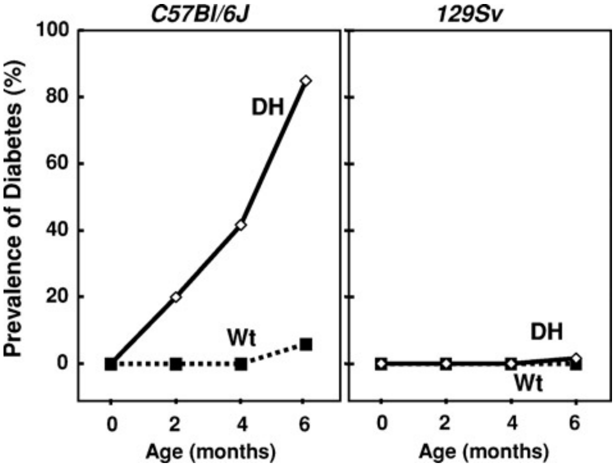

**FIGURE 8**
Incidence of diabetes with increasing age in wildtype (Wt) and IR/IRS-1 doubleheterozygous (DH) knockout mice on the backgrounds of C57Bl/6J (B6) and 129Sv.

etiology, genetically programmed insulin resistance, a delayed age of onset and a biphasic relationship between insulin and glucose levels. A genome-wide scan of the DH intercross mice has been performed using 90 polymorphic markers with an average distance of 20 cM. The results suggest that a locus on chromosome 12 is linked to hyperglycemia and a locus on chromosome 14 is significantly linked to hyperinsulinemia (Almind et al., manuscript submitted).

### Improved Insulin Tolerance in Triple Heterozygous Knockouts (IR/IRS-1/p85)

Recently, other studies have been extended to produce even more complex compound heterozygous animals. For example, mice with three partial defects in insulin signaling (IR/IRS-1/IRS-2 or IR/IRS-1/p85) have been created [63]. The phenotypes of these mice illustrate some of the complexity of polygenic disease. Thus, the IR/IRS-1/IRS-2 triple heterozygote mouse has severely impaired glucose tolerance and a doubling of the incidence of diabetes compared to the double heterozygotes. By contrast, the IR/IRS-1/p85 knockout is less severely affected than the IR/IRS-1 (±) mouse. In fact, heterozygosity for the p85 allele seems to protect mice from becoming diabetic [64]. This is demonstrated by the improved intraperitoneal insulin tolerance in p85 heterozygous mice as compared to wildtype mice (Figure 9*A*), as well as improved insulin tolerance in the triple heterozygous knockouts (IR/IRS-1/p85) as compared to the double heterozygous (IR/IRS-1) knockouts (Figure 9*B*). This may be due to an abundance of p85 subunit over p110 (the catalytic subunit) in wildtype mice, and thereby a competition between p85 monomer and the p85-p110 dimer causing an ineffective signaling. Thus, a reduction of p85 results in more efficient signaling and thus p85 may represent a novel therapeutic target for enhancing insulin signaling [65].

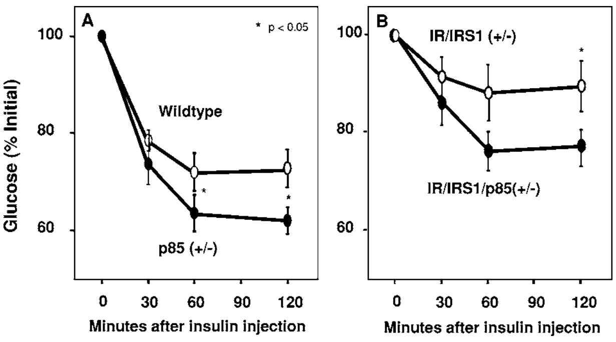

**FIGURE 9**
Increased insulin sensitivity in IR/IRS1/p85(±) mice. Insulin tolerance test were performed on 6-month-old male mice of the indicated genotypes. Results represent the blood glucose concentration as a percentage of the starting glucose value and are expressed as mean ± SEM [66].

### SUMMARY AND CONCLUSIONS

The painstaking process of generating constitutive and conditional knockout mice has paid off handsomely. The roles of the insulin receptor and its intracellular substrates in insulin action has been established and begun to shed light onto some of the proteins less obvious functions. We have learned how genetic predisposition plays itself out in the oligogenic and heterogeneous pathogenesis of type 2 diabetes and how the balance of proteins can affect the efficiency of signaling both positively and negatively. The IRS knockout mice have taught us how these proteins provide unique and complementary signals in insulin action. From the tissue specific knockouts we have learned that different tissues contribute uniquely to the pathogenesis of type 2 diabetes, but not always in the predicted way; that insulin resistance at different levels in the same tissue may produce different phenotypes; that tissues possess mechanisms of communication such that resistance in one tissue affects insulin signaling or metabolism in others; and that insulin has important effects in tissues not previously considered insulin responsive, including the brain and $\beta$-cells. The result of this work has led us to develop new hypotheses about the nature of the insulin action network.

### REFERENCES

[1] Virkamaki, A., Ueki, K., and Kahn, C. R. (1999) Protein-protein interaction in insulin signaling and the molecular mechanisms of insulin resistance. *J. Clin. Invest.,* **103,** 931–943.
[2] White, M. F. (1998) The IRS-signalling system: A network of docking proteins that mediate insulin action. *Mol. Cell. Biochem.,* **182,** 3–11.
[3] Sun, X. J., Wang, L. M., Zhang, Y., Yenush, L., Myers, M. G., Jr., Glasheen, E. M., Lane, W. S., Pierce, J. H., and White, M. F. (1995) Role of IRS-2 in insulin and cytokine signalling. *Nature,* **377,** 173–177.
[4] Holgado-Madruga, M., Emlet, D. R., Moscatello, D. K., Godwin, A. K., and Wong, A. J. (1996) A Grb2-associated docking protein in EGF- and insulin-receptor signalling. *Nature,* **379,** 560–563.
[5] Kraegen, E. W., Lazarus, L., and Campbell, L. V. (1983) Failure of insulin infusion during euglycemia to influence endogenous basal insulin secretion. *Metabolism,* **32,** 622–627.
[6] Lavan, B. E., Fantin, V. R., Chang, E. T., Lane, W. S., Keller, S. R., and Lienhard, G. E. (1997) A novel 160 kDa phosphotyrosine protein in insulin-treated embryonic kidney cells is a new member of the insulin receptor substrate family. *J. Biol. Chem.,* **272,** 21403–21407.
[7] Lee, C. H., Li, W., Nishimura, R., Zhou, M., Batzer, A. G., Myers, M. G., Jr., White, M. F., Schlessinger, J., and Skolnik, E. Y. (1993) Nck associates with the SH2 domain docking proteins IRS-1 in insulin stimulated cells. *Proc. Natl. Acad. Sci. U.S.A.,* **90,** 11713–11717.

[8] Beitner-Johnson, D., Blakesley, V. A., Shen-Orr, Z., Jimenez, M., Stannard, B., Wang, L. M., Pierce, J. H., and LeRoith, D. (1996) The proto-oncogene product c-Crk associates with insulin receptor substrate-1 and 4PS. *J. Biol. Chem.,* **271,** 9287–9290.

[9] Cheatham, B., Vlahos, C. J., Cheatham, L., Wang, L., Blenis, J., and Kahn, C. R. (1994) Phosphatidylinositol 3-kinase activation is required for insulin stimulation of pp70 S6 kinase, DNA synthesis, and glucose transporter translocation. *Mol. Cell. Biol.,* **14,** 4902–4911.

[10] Skolnik, E. Y., Batzer, A. G., Li, N., Lee, C. H., Lowenstein, E. J., Mohammadi, M., Margolis, B., and Schlessinger, J. (1993) The function of GRB2 in linking the insulin receptor to ras signaling pathways. *Science,* **260,** 1953–1955.

[11] Capecchi, M. R. (2001) Generating mice with targeted mutations. *Nat. Med.,* **7,** 1086–1090.

[12] Araki, E., Lipes, M. A., Patti, M. E., Brüning, J. C., Haag, B. L., III, Johnson, R. S., and Kahn, C. R. (1994) Alternative pathway of insulin signaling in mice with targeted disruption of the IRS-1 gene. *Nature,* **372,** 186–190.

[13] Tamemoto, H., Kadowaki, T., Tobe, K., Yagi, T., Sakura, H., Hayakawa, T., Terauchi, Y., Ueki, K., Kaburagi, Y., Satoh, S., *et al.* (1994) Insulin resistance and growth retardation in mice lacking insulin receptor substrate-1. *Nature,* **372,** 182–186.

[14] Withers, D. J., Gutierrez, J. S., Towery, H., Burks, D. J., Ren, J. M., Previs, S., Zhang, Y., Bernal, D., Pons, S., Shulman, G. I., Bonner-Weir, S., and White, M. F. (1998) Disruption of IRS-2 causes type 2 diabetes in mice. *Nature,* **391,** 900–904.

[15] Liu, S. C., Wang, Q., Lienhard, G. E., and Keller, S. R. (1999) Insulin receptor substrate 3 is not essential for growth or glucose homeostasis. *J. Biol. Chem.,* **274,** 18093–18099.

[16] Fantin, V. R., Wang, Q., Lienhard, G. E., and Keller, S. R. (2000) Mice lacking insulin receptor substrate 4 exhibit mild defects in growth, reproduction, and glucose homeostasis. *Am. J. Physiol. Endocrinol. Metab.,* **278,** E127–E133.

[17] Reaven, G. M. (1987) Non-insulin-dependent diabetes mellitus, abnormal lipoprotein metabolism and atherosclerosis. *Metabolism,* **36,** 1–8.

[18] Kulkarni, R. N., Winnay, J. N., Daniels, M., Bruning, J. C., Flier, S. N., Hanahan, D., and Kahn, C. R. (1999) Altered function of insulin receptor substrate-1-deficient mouse islets and cultured beta-cell lines. *J. Clin. Invest.,* **104,** R69–R75.

[19] Taylor, S. I. (1992) Lilly Lecture: Molecular mechanisms of insulin resistance-Lessons from patients with mutations in the insulin receptor gene. *Diabetes,* **41,** 1473–1490.

[20] Accili, D. (1995) Molecular defects of the insulin receptor gene. *Diab. Metab. Rev.,* **11,** 47–62.

[21] Accili, D., Drago, J., Lee, E. J., Johnson, M. D., Cool, M. H., Salvatore, P., Asico, L. D., Jose, P. A., Taylor, S. I., and Westphal, H. (1996) Early neonatal death in mice homozygous for a null allele of the insulin receptor gene. *Nat. Genet.,* **12,** 106–109.

[22] Louvi, A., Accili, D., and Efstratiadis, A. (1997) Growth-promoting interaction of IGF-II with the insulin receptor during mouse embryonic development. *Dev. Biol.,* **189,** 33–48.

[23] Duvillie, B., Cordonnier, N., Deltour, L., Dandoy-Dron, F., Itier, J. M., Monthioux, E., Jami, J., Joshi, R. L., and Bucchini, D. (1997) Phenotypic alterations in insulin-deficient mutant mice. *Proc. Natl. Acad. Sci. U.S.A.,* **94,** 5137–5140.

[24] Di Cola, G., Cool, M. H., and Accili, D. (1997) Hypoglycemic effect of insulin-like growth factor-1 in mice lacking insulin receptors. *J. Clin. Invest.,* **99,** 2538–2544.

[25] Gu, H., Marth, J. D., Orban, P. C., Mossmann, H., and Rajewsky, K. (1994) Deletion of a DNA polymerase beta gene segment in T cells using cell type-specific gene targeting. *Science,* **265,** 103–106.

[26] Ferrannini, E., Galvan, A. Q., Gastaldelli, A., Camastra, S., Sironi, A. M., Toschi, E., Baldi, S., Frascerra, S., Monzani, F., Antonelli, A., Nannipieri, M., Mari, A., Seghieri, G., and Natali, A. (1999) Insulin: New roles for an ancient hormone. *Eur. J. Clin. Invest.,* **29,** 842–852.

[27] Cline, G. W., Petersen, K. F., Krssak, M., Shen, J., Hundal, R. S., Trajanoski, Z., Inzucchi, S., Dresner, A., Rothman, D. L., and Shulman, G. (1999) Impaired glucose transport as a cause of decreased insulin-stimulated muscle glycogen synthesis in type 2 diabetes. *N. Engl. J. Med.,* **341,** 240–246.

[28] Kahn, C. R. (1994) Insulin action, diabetogenes, and the cause of type II diabetes (Banting Lecture) *Diabetes,* **43,** 1066–1084.

[29] Bruning, J. C., Michael, M. D., Winnay, J. N., Hayashi, T., Horsch, D., Accili, D., Goodyear, L. J., and Kahn, C. R. (1998) A muscle-specific insulin receptor knockout exhibits features of the metabolic syndrome of NIDDM without altering glucose tolerance. *Mol. Cell.,* **2,** 559–569.

[30] Wojtaszewski, J. F. P., Higaki, Y., Dufresne, S., Hirshman, M. F., Michael, M. D., and Goodyear, L. J. (1999) Exercise increases glucose transport and insulin action in muscle ionsulin receptor knockout (MIRKO) mice. *Diabetes,* **48,** A14 (Abstr.)

[31] Baron, A. D. (1994) Hemodynamic actions of insulin. *Am. J. Physiol.,* **267,** E187–E202.

[32] Etgen, G. J., Jr., Fryburg, D. A., and Gibbs, E. M. (1997) Nitric oxide stimulates skeletal muscle glucose transport through a calcium/contraction- and phosphatidylinositol-3-kinase-independent pathway. *Diabetes,* **46,** 1915–1919.

[33] Young, M. E., Radd, G. K., and Leighton, B. (1997) Nitric oxide stimulates glucose transport and metabolism in rat skeletal muscle *in vitro*. *Biochem. J.* **322,** 223–228.

[34] Golay, A., DeFronzo, R. A., Ferrannini, E., Simonson, D. C., Thorin, D., Acheson, K., Thiebaud, D., Curchod, B., Jequier, E., and Felber, J. P. (1988) Oxidative and non-oxidative glucose metabolism in non-obese type 2 (non-insulin-dependent) diabetic patients. *Diabetologia,* **31,** 585–591.

[35] Marin, P., Hogh-Kristiansen, I., Jansson, S., Krotkiewski, M., Holm, G., and Bjorntorp, P. (1992) Uptake of glucose carbon in muscle glycogen and adipose tissue triglycerides in vivo in humans. *Am. J. Physiol.,* **263,** E473–E480.

[36] Rosen, E. D., Walkey, C. J., Puigserver, P., and Spiegelman, B. M. (2000) Transcriptional regulation of adipogenesis. *Genes Dev.,* **14,** 1293–1307.

[37] Bluher, M., Michael, M. D., Peroni, O. D., Ueki, K., Carter, N., Kahn, B. B., and Kahn, C. R. (2002) Adipose Tissue Selective Insulin Receptor Knockout Protects against Obesity and Obesity-Related Glucose Intolerance. *Dev. Cell,* **3,** 25–38.

[38] Larsen, P. L., Albert, P. S., and Riddle, D. L. (1995) Genes that regulate both development and longevity in Caenorhabditis elegans. *Genetics,* **139,** 1567–1583.

[39] Rogina, B., Reenan, R. A., Nilsen, S. P., and Helfand, S. L. (2000) Extended life-span conferred by cotransporter gene mutations in Drosophila. *Science,* **290,** 2137–2140.

[40] Coschigano, K. T., Clemmons, D., Bellush, L. L., and Kopchick, J. J. (2000) Assessment of growth parameters and life span of GHR/BP gene-disrupted mice. *Endocrinology,* **141,** 2608–2613.
[41] Lowell, B. B., Susulic, V., Hamann, A., Lawitts, J. A., Himms-Hagen, J., Boyer, B. B., Kozak, L. P., and Flier, J. S. (1993) Development of obesity in transgenic mice after genetic ablation of brown adipose tissue. *Nature,* **366,** 740–742.
[42] Guerra, C., Navarro, P., Valverde, A. M., Arribas, M., Bruning, J., Kozak, L. P., Kahn, C. R., and Benito, M. (2001) Brown adipose tissue-specific insulin receptor knockout shows diabetic phenotype without insulin resistance. *J. Clin. Invest.,* **108,** 1205–1213.
[43] Cherrington, A. D. (1999) Banting Lecture 1997. Control of glucose uptake and release by the liver in vivo. *Diabetes,* **48,** 1198–1214.
[44] Michael, M. D., Kulkarni, R. N., Postic, C., Previs, S. F., Shulman, G. I., Magnuson, M. A., and Kahn, C. R. (2000) Loss of insulin signaling in hepatocytes leads to severe insulin resistance and progressive hepatic dysfunction. *Molecular Cell,* **6,** 87–97.
[45] Postic, C., Shiota, M., Niswender, K. D., Jetton, T. L., Chen, Y., Moates, J. M., Shelton, K. D., Lindner, J., Cherrington, A. D., and Magnuson, M. A. (1999) Dual roles for glucokinase in glucose homeostasis as determined by liver and pancreatc $\beta$ cell-specific gene knock-outs using Cre recombinase. *J. Biol. Chem.,* **274,** 305–315.
[46] Kuboki, K., Jiang, Z. Y., Takahara, N., Ha, S. W., Igarashi, M., Yamauchi, T., Feener, E. P., Herbert, T. P., Rhodes, C. J., and King, G. L. (2000) Regulation of endothelial constitutive nitric oxide synthase gene expression in endothelial cells and in vivo: A specific vascular action of insulin. *Circulation,* **101**(6), 676–681.
[47] Zeng, G., Nystrom, F. H., Ravichandran, L. V., Cong, L. N., Kirby, M., Mostowski, H., and Quon, M. J. (2000) Roles for insulin receptor, PI3-kinase, and Akt in insulin-signaling pathways related to production of nitric oxide in human vascular endothelial cells. *Circulation,* **101**(13), 1539–1545.
[48] Polonsky, K. S., Sturis, J., and Bell, G. I. (1996) Non-insulin dependent diabetes mellitus—A genetically programmed failure of the beta cell to compensate for insulin resistance. *N. Engl. J. Med.,* **334,** 777–783.
[49] Matschinsky, F. M. (1996) Banting Lecture, 1995: A lesson in metabolic regulation inspired by the glucokinase glucose senor paradigm. *Diabetes,* **45,** 223–241.
[50] Leibiger, B., Leibiger, I. B., Moede, T., Kemper, S., Kulkarni, R. N., Kahn, C. R., de Vargas, L. M., and Berggren, P. O. (2001) Selective insulin signaling through A and B insulin receptors regulates transcription of insulin and glucokinase genes in pancreatic beta cells. *Mol. Cell.,* **7,** 559–570.
[51] Kulkarni, R. N., Bruning, J. C., Winnay, J. N., Postic, C., Magnuson, M. A., and Kahn, C. R. (1999) Tissue-specific knockout of the insulin receptor in pancreatic $\beta$ cells creates an insulin secretory defect similar to that in Type 2 diabetes. *Cell,* **96,** 329–339.
[52] Hirayama, I., Tamemoto, H., Yokota, H., Kubo, S., Wang, J., Kuwano, H., Nagamachi, Y., Takeuchi, T., and Izumi, T. (1999) Insulin receptor-related receptor is expressed in pancreatic beta-cells and stimulates tyrosine phosphorylation of insulin receptor substrate-1 and -2. *Diabetes,* **48,** 1237–1244.
[53] Kitamura, T., Kido, Y., Nef, S., Merenmies, J., Parada, L. F., and Accili, D. (2001) Preserved pancreatic beta-cell development and function in mice lacking the insulin receptor-related receptor. *Mol. Cell. Biol.,* **21,** 5624–5630.
[54] Havrankova, J., Roth, J., and Brownstein, M. (1978) Insulin receptors are widely distributed in the central nervous system of the rat. *Nature,* **272,** 827–829.
[55] Bruning, J. C., Gautam, D., Burks, D. J., Gillette, J., Schubert, M., Orban, P. C., Klein, R., Krone, W., Muller-Wieland, D., and Kahn, C. R. (2000) Role of brain insulin receptor in control of body weight and reproduction. *Science,* **289,** 2122–2125.
[56] Obici, S., Feng, Z., Karkanias, G., Baskin, D. G., and Rossetti, L. (2002) Decreasing hypothalamic insulin receptors causes hyperphagia and insulin resistance in rats. *Nat Neurosci.,* **5,** 566–572.
[57] Thorell, A., Hirshman, M. F., Nygren, J., Jorfeldt, L., Wojtaszewski, J. F., Dufresne, S. D., Horton, E. S., Ljungqvist, O., and Goodyear, L. J. (1999) Exercise and insulin cause GLUT-4 translocation in human skeletal muscle. *Am. J. Physiol.,* **277,** E733–E741.
[58] Zisman, A., Peroni, O. D., Abel, D., Michael, M. D., Mauvais-Jarvis, F., Lowell, B. B., Wojtaszewski, J. F. P., Hirshman, M. F., Virkamaki, A., Goodyear, L. J., Kahn, C. R., and Kahn, B. B. (2000) Targeted disruption of the glucose transporter 4 selectively in muscle causes insulin resistance and glucose intolerance. *Nature Medicine,* **6,** 924–928.
[59] Kim, J. K., Zisman, A., Fillmore, J. J., Peroni, O. D., Kotani, K., Perret, P., Zong, H., Kahn, C. R., Kahn, B. B., and Shulman, G. I. (2001) Glucose toxicity and the development of diabetes in mice with muscle-specific inactivation of GLUT4. *J. Clin. Invest.,* **108,** 153–160.
[60] Abel, E. D., Peroni, O., Kim, J. K., Kim, Y. B., Boss, O., Hadro, E., Minnemann, T., Shulman, G. I., and Kahn, B. B. (2001) Adipose-selective targeting of the GLUT4 gene impairs insulin action in muscle and liver. *Nature,* **409,** 729–733.
[61] Bruning, J. C., Winnay, J., Bonner-Weir, S., Taylor, S. I., Accili, D., and Kahn, C. R. (1997) Development of a novel polygenic model of NIDDM in mice heterozygous for IR and IRS-1 null alleles. *Cell,* **88,** 561–572.
[62] DeFronzo, R. A. (1992) Pathogenesis of type 2 (non-insulin-dependent) diabetes mellitus: A balanced overview. *Diabetologia,* **35,** 389–397.
[63] Kido, Y., Burks, D. J., Withers, D., Bruning, J. C., Kahn, C. R., White, M. F., and Accili, D. (2000) Tissue-specific insulin resistance in mice with mutations in the insulin receptor, IRS-1, and IRS-2. *J. Clin. Invest.,* **105,** 199–205.
[64] Mauvais-Jarvis, F., Ueki, K., Fruman, D. A., Hirshman, M. F., Sakamoto, K., Goodyear, L. J., Iannacone, M., Accili, D., Cantley, L. C., and Kahn, C. R. (2002) Reduced expression of the murine p85alpha subunit of phosphoinositide 3-kinase improves insulin signaling and ameliorates diabetes. *J. Clin. Invest.,* **109,** 141–149.
[65] Ueki, K., Fruman, D. A., Brachmann, S. M., Tseng, Y. H., Cantley, L. C., and Kahn, C. R. (2002) Molecular balance between the regulatory and catalytic subunits of phosphoinositide 3-kinase regulates cell signaling and survival. *Mol. Cell. Biol.,* **22,** 965–977.
[66] Mauvais-Jarvis, F., Ueki, K., Fruman, D. A., Hirshman, M. F., Sakamoto, K., Goodyear, L. J., Iannacone, M., Accili, D., Cantley, L. C., and Kahn, C. R. (2002) Reduced expression of the murine p85alpha subunit of phosphoinositide 3-kinase improves insulin signaling and ameliorates diabetes. *J. Clin. Invest.,* **109,** 141–149.

